# Root, Nodule and Soil Bacterial Communities Associated With the Invasive Nitrogen‐Fixing *Lupinus polyphyllus*

**DOI:** 10.1002/ece3.70669

**Published:** 2024-12-05

**Authors:** Satu Ramula, Seyed Abdollah Mousavi, Eero J. Vesterinen

**Affiliations:** ^1^ Department of Biology University of Turku Turku Finland

**Keywords:** endophyte, microbiota, plant bacterial community, plant invasion, rhizobia, soil bacterial community

## Abstract

Plants host microorganisms that can facilitate their success in becoming invasive. Established plant invasions might thus provide useful insights into potential changes in plant‐associated microbiomes over the course of the invasion process. Here, we investigated the endophytic bacterial communities of the invasive herbaceous legume *Lupinus polyphyllus* , which is able to form mutualistic associations with N‐fixing bacteria. More specifically, we examined the alpha diversity (observed bacterial taxa richness and Shannon diversity) and composition of bacterial communities in roots and nodules sampled from core and edge locations within 10 established invasion sites (> 10 years old) in southwestern Finland. Moreover, we compared the alpha diversity and structure of bacterial communities in the rhizosphere and bulk soil between core and edge locations within these invasion sites. We found that roots and nodules had distinctive endophytic bacterial communities, with roots having 24% higher bacterial alpha diversity (Shannon diversity) than nodules. In nodules, the dominant bacteria were assigned to the family *Bradyrhizobiaceae*, which includes N‐fixing bacteria. Soil bacterial communities, instead, were shaped by soil type, with bulk soil hosting up to 27% higher alpha diversity (richness and Shannon diversity) than rhizosphere soil; however, there was no apparent difference in their community composition. Soil bacterial communities were only weakly associated with soil chemistry. Endophytic and soil bacterial communities did not differ between core and edge locations within the established invasions. Our findings suggest that *L. polyphyllus* may not induce dramatic changes in the bacterial communities with which it associates over the course of the local invasion process.

## Graphical abstract

We found that roots and nodules of the invasive herb *Lupinus polyphyllus* had distinctive endophytic bacterial communities, but endophytic and soil bacterial communities did not differ between core and edge locations within the established invasions. Our findings suggest that *L. polyphyllus* may not induce dramatic changes in the bacterial communities with which it associates over the course of the local invasion process.

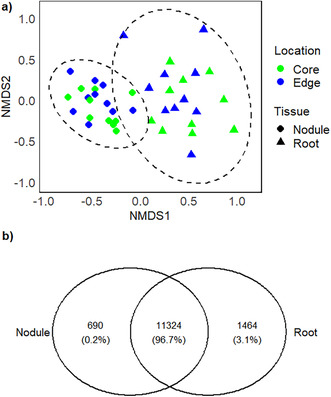

## 1 Introduction

Collectively, all of the microorganisms associated with an individual plant form the plant microbiome, which can often affect plant performance. Interactions between plants and soil microbes can be critical to plant establishment and invasiveness (Parker 2001; Dawson and Schrama 2016) due to the ability of some microbes to promote plant growth and mitigate abiotic stress (Bulgarelli et al. 2013; Begum et al. 2019; Trivedi et al. 2022), thus potentially contributing to invasion success (Rout et al. 2013). In particular, endophytic microbes that inhabit the internal plant tissues are likely to be beneficial to the host plant (Compant, Clément, and Sessitsch 2010). As an example, mutualistic partners such as N‐fixing bacteria (rhizobia) and mycorrhizal fungi assist in nutrient uptake in nutrient‐poor soils (Smith and Smith 2011; Bulgarelli et al. 2013) and may provide a competitive advantage to their host plant over coexisting, non‐mutualistic plant species (Rout et al. 2013).

Plants are able to modify the structure and function of their microbiomes, for example, by enriching beneficial microbes (Reinhold‐Hurek et al. 2015), as well as to alter soil chemistry (Gibbons et al. 2017). For terrestrial plants, bulk soil (i.e., the soil beyond the direct influence of plant roots) serves as a reservoir for microorganisms. Plants use root exudates to attract beneficial microbes from the bulk soil to the rhizosphere (i.e., the soil compartment influenced by rhizodeposits) and then to the roots (Compant, Clément, and Sessitsch 2010). As a consequence, microbial diversity tends to be lower towards the inside of the root (i.e., the dilution effect), with root endophytic microbial communities consisting of a few dominant phyla (Bulgarelli et al. 2013; Wang et al. 2020). Plant microbiomes can also be species‐specific (Reinhold‐Hurek et al. 2015) and may differ among plant organs or tissue types (Trivedi et al. 2020).

So far, most research has focused on the effects of invasive alien plants on soil microbiomes by comparing invaded and uninvaded rhizosphere soils (reviewed in Custer and van Diepen 2020; Torres et al. 2021), while the endophytic microbiomes of plant invaders have received less attention (but see Birnbaum et al. 2016; Lu‐Irving et al. 2019; Jara‐Servin et al. 2023). Moreover, little is known about local changes in plant endophytic microbiomes over the course of plant invasions. Previous studies have reported that invasive alien plants can undergo rapid evolution in their introduced range (Buswell, Moles, and Hartley 2011; Colautti and Barrett 2013; Clements and Jones 2021), exhibiting different phenotypic traits between core regions and invasion fronts (Colautti and Barrett 2013). Such phenotypic shifts in plant invaders might also be reflected in their microbiomes, which are typically expected to change within the introduced range over time (Lu‐Irving et al. 2019). However, for short‐lived plant invaders, changes in microbiomes might occur even within local invasions, in which more established core locations have a longer invasion history than the expanding edges (Batten et al. 2006). Therefore, established plant invasions might provide useful insights into changes in plant‐associated microbiomes over the course of the invasion process.

Here, we focused on the bacterial communities associated with the short‐lived perennial herb *Lupinus polyphyllus* Lindl. (garden lupin, Fabaceae). The species is a summer‐green herb, 50–100 cm tall, that has a strong taproot with some side roots (Eckstein et al. 2023). It originates from western North America and is considered invasive on many continents, including Europe (Eckstein et al. 2023). As a legume, it can form a mutualistic association with N‐fixing bacteria belonging to the genus *Bradyrhizobium* (Stepkowski et al. 2007; Stępkowski et al. 2018; Ramula, Mousavi, and Kalske 2023). The species inhabits diverse habitat types, from mountain meadows in central Europe to nutrient‐poor road verges and wastelands in Fennoscandia (Eckstein et al. 2023). It is classified as one of the most harmful plant invaders in Europe (Nentwig et al. 2018), partly due to its associations with N‐fixing bacteria which lead to an increase in soil N content (Hiltbrunner et al. 2014; Prass et al. 2022). In its introduced range, *L. polyphyllus* is associated with changes in plant community composition and declines in the diversity of vascular plant species and arthropods (Eckstein et al. 2023). Moreover, the species contains numerous chemical compounds (alkaloids) that may have negative allelopathic effects on co‐existing plant species (Eckstein et al. 2023). In our study area in southwestern Finland, the invasion history of the species dates back to the late 1800s when it was reported to have escaped from gardens (Eckstein et al. 2023). In this study, we examined the alpha diversity and composition of endophytic bacterial communities in roots and root nodules sampled from core and edge locations within established invasion sites (> 10 years old). We also compared the alpha diversity and structure of bacterial communities in the rhizosphere and in bulk soil between core and edge locations within invasion sites and explored differences in the chemistry of rhizosphere soil (nutrient content, pH) between the two locations. We hypothesised that: (1) the alpha diversity and community composition of endophytic bacteria would differ between tissue types (roots, nodules) and between sampling locations (core, edge) within invasions, with diversity being higher in roots than in nodules, and N‐fixing bacteria (members of *Bradyrhizobiaceae*) dominating in nodules sampled from core locations in particular; (2) soil bacterial alpha diversity would be higher in bulk soil than in the rhizosphere due to the dilution effect (Reinhold‐Hurek et al. 2015); (3) soil bacterial communities would be associated with soil chemistry; and (4) soil nitrogen content would be higher in core than in edge locations within established invasions due to their longer invasion history and a higher relative abundance of rhizobia associated with the study species.

## 2 Materials and Methods

### 2.1 Root and Soil Sampling

We carried out root and soil sampling in 10 sites with established invasions of *L. polyphyllus* over 4 days (29 June–2 July) in the summer of 2020 when the species was flowering. All of these sites were close to Turku in southwestern Finland (latitude: 60.357–60.521, longitude: 22.168–22.740; distances 1.7–32.7 km apart). They featured sandy moraine or clay soil and were located in wastelands, road verges, former fields, or forest understoreys. The exact age of the invasions is unknown, but the species has been present in each location since at least 2010 when we visited the populations for the first time (Ramula 2014). The size of these invasions varies between 65 and 2400 m^2^ (mean ± SD = 617.4 ± 736.4 m^2^), and currently, they contain hundreds of individuals (mean cover = 59.7%, range: 2%–96%). In each invasion site, we haphazardly sampled 10 flowering individuals from the core and edge locations, respectively, with plant density typically being lower in the latter (mean ± SD = 3.3 ± 1.1 for core and 2.0 ± 0.9 for edge). We dug up each sampled plant, used a spade to gently remove soil close to its root system, cut a ca. 10‐cm piece of root containing nodules and placed it in a plastic ziplock bag, resulting in a total of 200 root samples (10 plants × 2 locations × 10 invasion sites). In addition, we collected soil samples from the ectorhizosphere (hereafter rhizosphere) of three plants (at a depth of 10–15 cm) and bulk soil (at a depth of > 20 cm) in the core and edge locations; these samples were pooled within each location per invasion site and stored in a plastic bag. Altogether, we had 40 soil samples (2 locations (core and edge) × 2 soil types (rhizosphere and bulk) × 10 invasion sites). To avoid cross‐contamination, the spade and secateurs were wiped with ethanol after each sampling event. We transported all samples in a cold box to the laboratory and preserved the soil samples at −80°C for later use. In the laboratory, we rinsed the roots with tap water to remove the soil and cut about 2 cm of each sample and some nodules for further analysis. These root and nodule samples were surface‐sterilised in 96% ethanol for 1 min and 3% NaClO for 3–5 min and rinsed with deionised water three times. The specimens were preserved in Eppendorf tubes at −80°C for DNA isolation.

To characterise soil chemistry, we collected separate soil samples from the rhizosphere of *L. polyphyllus* in the core and edge locations of each invasion site (no bulk soil was sampled). Again, we sampled three flowering plants per location and pooled their rhizosphere soil (0.5 L soil in total), resulting in 20 soil samples (2 locations × 10 invasion sites). The following soil properties were analysed: total nitrogen (N), nitrate (NO_3_^−^), ammonium (NH_4_^+^), calcium (Ca), phosphorus (P), potassium (K), magnesium (Mg) and pH. The soil analysis was carried out by Eurofins Agro in Finland (https://www.eurofins.fi/agro).

### 2.2 DNA Extraction

We cut the root samples into pieces 2–4 mm in length using sterilised (in 70% ethanol) blades. We then crushed the nodules and root pieces (up to 150 mg) using tweezers sterilised in 70% ethanol for DNA extraction. For the soil samples, we used 300 mg of each sample for DNA extraction. For all samples (roots, nodules, soil), we extracted DNA with the NucleoSpin Soil Kit (Macherey‐Nagel GmbH Co. KG, Duren, Germany) following the provided protocol. To improve the DNA yield, root and nodule samples were placed in TissueLyser II (Qiagen) for the sample lysis step. The extracted DNA samples were preserved at −20°C for PCR and sequencing.

### 2.3 Polymerase Chain Reaction (PCR)

Locus‐specific PCR and library preparation was done by the company Bioname Ltd., Finland (www.bioname.fi). The ribosomal bacterial 16S v8–v9 gene region was amplified using nested PCR and two primer pairs (799F [AACMGGATTAGATACCCKG; Chelius and Triplett 2001] and 1492R [GGYTACCTTGTTACGACTT; Lane 1991]), and (1062F [GTCAGCTCGTGYYGTGA; modified from Ghyselinck et al. 2013] and 1390R [ACGGGCGGTGTGTRCAA; modified from Zheng et al. 1996]). The secondary pair of the primers included a linker‐tag to enable the subsequent attachment of NGS adapters. To increase amplicon library diversity, the secondary primer pair was used as four different versions so that they included heterogeneity spacers between the linker‐tag and the locus‐specific oligo. A blank PCR control was added to each PCR batch to evaluate the purity of reagents and the level of cross‐contamination. In this project, the PCR controls were done in parallel in separate tubes because the DNA plates contained no empty wells. Initially, we carried out a pre‐PCR using the primers 799F and 1492R to amplify a longer fragment of the bacterial 16S region. This primer pair is used to exclude plant chloroplast amplification (Chelius and Triplett 2001). The reaction setup followed Kankaanpää et al. (2020) and included 5 μL of 2 × MyTaq HS Red Mix (Bioline, UK), 2.4 μL of H_2_O, 150 nM of each primer and 2 μL of DNA extract per sample in a 10‐μL volume. Cycling conditions for 799F and 1492R were as follows: 3 min at 95°C, 35 cycles of 45 s at 95°C, 45 s at 54°C, and 1 min at 72°C, ending with 5 min at 72°C. The nested PCR reaction included the locus‐specific primer pair 1062F and 1390R. The PCR mixture from the pre‐PCR round was used as a DNA template in this round of PCR. All PCR reactions after the pre‐PCR were carried out in two technical replicates, in which each replicate contained two heterogeneity versions of each primer.

### 2.4 Library Preparation and Sequencing

Library PCR followed, with minor modifications, the procedure of Vesterinen et al. (2016). We used a dual indexing strategy, in which each reaction (including technical replicates) was prepared with a unique combination of forward and reverse indices. All index sets were balanced perfectly so that each nucleotide position included either T/G or A/C, which ensures base calling for each channel during sequencing. For a reaction volume of 10 μL, we mixed 5 μL of MyTaq HS RedMix, 500 nM of each tagged and indexed primer (i7 and i5) and 3 μL of locus‐specific PCR product from the first PCR phase. For library preparation PCR, we used the following protocol: initial denaturation for 3 min at 98°C, then 12 cycles of 20 s at 95°C, 15 s at 60°C and 30 s at 72°C, followed by 3 min at 72°C. All the indexed reactions were then pooled and purified using magnetic beads (Vesterinen et al. 2018). Sequencing was done at the Turku Centre for Biotechnology (Turku, Finland), on an Illumina NovaSeq6000 SP FlowCell v1.5 PE 2 × 250 apparatus (Illumina Inc., San Diego, California, USA), run together with other samples, including a PhiX control library.

### 2.5 Bioinformatics and Diversity Measures

Our bioinformatics pipeline closely followed that in Kaunisto et al. (2020) as summarised below. In this study, we processed a total of 44,489,226 raw reads (25,151,901 in PCR replicate 1 and 19,337,325 in PCR replicate 2). Paired‐end reads were merged and trimmed for quality using 64‐bit VSEARCH version 2.14.2 (Rognes et al. 2016), and the primers were removed from the merged reads using the software CUTADAPT version 2.7 (Martin 2011) with 20% rate for primer mismatches and strict length parameters: minimum length 285 bp, maximum length 320 bp (longer sequences clipped from 3′ end). After removing singletons and chimers, the unique reads were clustered into ZOTUs (zero‐radius operational taxonomic units) with 32‐bit USEARCH version 11 (Edgar 2010). ZOTUs were mapped back to the primer‐trimmed reads to construct a zotu table with the ‘usearch_global’ algorithm in VSEARCH (Rognes et al. 2016). The resulting sequence variants were assigned to taxa using custom databases with SINTAX (Edgar 2010) in VSEARCH (Rognes et al. 2016). The databases were downloaded from https://drive5.com/usearch/manual/sintax_downloads.html with the following reference: 16S RDP training set rdp_16s_v18 (21 k seqs). The reads from both PCR replicates were pooled if reads were detected in both of them. We removed singletons (read count < 2) and only retained reads assigned as Bacteria.

Bioinformatic processing resulted in 15,003 ZOTUs for subsequent analyses. Since the sampling design for soil differed from that for roots and nodules, we analysed these two data sets separately. After rarefying (8104 sequences per sample for roots and nodules and 88 sequences for soil, Figure S1), we continued our analyses with a total of 13,478 ZOTUs for the root and nodule samples, and 1141 ZOTUs for the soil samples. Low sequence counts for soil were due to the inadequate dilution of the samples. To evaluate the diversity of these bacterial communities, we calculated two alpha diversity measures (observed ZOTU richness and Shannon index (H′)) separately for the root and nodule data and for the soil data using the R (R Core Team 2023) packages ‘phyloseq’ (McMurdie and Holmes 2013) and ‘microeco’ (Liu et al. 2021).

### 2.6 Statistical Analyses

To analyse the alpha diversity of plant endophytic bacteria, we conducted a linear mixed model (LMM, lmer4::lmer) for each diversity measure (observed ZOTU richness and Shannon diversity) with tissue type (root, nodule), sampling location (core, edge), and their interaction as fixed explanatory variables, and invasion site as a random factor. For the soil samples collected, we examined the effect of soil type (rhizosphere, bulk), location (core, edge), and their interaction on soil bacterial alpha diversity (ZOTU richness and Shannon diversity) with an LMM. Invasion site was again included as a random factor in the model. For the soil data, we repeated the analysis without four rhizosphere samples that had much lower alpha diversity than the other samples (see Figure 2b,d) and present the results from both analyses when they differ. To test whether the effect of sampling location on endophytic and soil bacterial alpha diversity differed across invasion sites, we fitted different slopes for each invasion site and compared the model AIC to that of the model with a constant slope. Different slopes were not supported in any of the cases (AIC was lower for the models with a constant slope). For all models, we evaluated the significance of the fixed variables with an *F* test based on the Kenward‐Roger method (lmerTest::anova; Kuznetsova, Brockhoff, and Christensen 2017). We verified assumptions visually from residual plots.

We explored differences in the composition of endophytic and soil bacterial communities with a permutational multivariate analysis of variance (PERMANOVA, vegan::adonis2; Oksanen et al. 2022) based on Bray‐Curtis dissimilarities calculated from the relative abundances of bacterial ZOTUs (separate analyses for the two data sets). For the analysis of plant bacterial communities, we pooled 10 replicates (plants) per location for each invasion site and used tissue type (root, nodule), location (core, edge) and their interaction as fixed explanatory variables. For the analysis of soil bacterial communities, we used soil type (rhizosphere, bulk), location (core, edge) and their interaction as fixed explanatory variables. For both analyses, we tested the significance of each factor with 9999 permutations. The results were visualised with non‐metric multidimensional scaling (NMDS), with the number of dimensions based on the lowest stress value obtained: two dimensions for the root bacterial communities (stress = 0.15) and three dimensions for the soil bacterial communities (stress = 0.19). Finally, we explored whether the variances of the endophytic and soil bacterial communities differed in relation to different grouping variables, including tissue type, soil type and location (vegan::betadisper). This analysis reveals potential differences in the magnitude of the variation between the bacterial communities compared.

For significant categorical variables in the PERMANOVAs above, we carried out indicator species analysis (indspecies::multipatt with func = IndVal.g; De Cáceres and Legendre 2009). This analysis explores differences in the abundance and frequency of each ZOTU in the samples collected from the different levels of categorical variables. Here, we used indicator species analysis to identify ZOTUs that were associated with roots and nodules. We visualised shared ZOTUs between roots and nodules with a Venn diagram (microeco::trans_venn).

To test whether the soil bacterial communities were associated with soil chemistry, we conducted a canonical corresponding analysis, CCA (vegan::cca) based on six uncorrelated soil variables estimated from the rhizosphere soil only (total *N*, NH_4_^+^, Ca, P, Mg, and pH). We did not consider K due to its strong correlation with Mg (*r* = 0.79) and *N* (*r* = 0.69) and also excluded NO_3_^−^ due to its correlation with total *N* (*r* = 0.99). The bacterial data were relative abundances and the soil variables were standardised to have unit variance. The significance of each soil variable was evaluated based on 9999 permutations. Finally, we analysed the effect of sampling location (core, edge) on soil abiotic properties with separate t‐tests conducted for each of the six soil variables (t.test function in R).

## 3 Results

### 3.1 Endophytic Bacterial Communities

**FIGURE 1:**
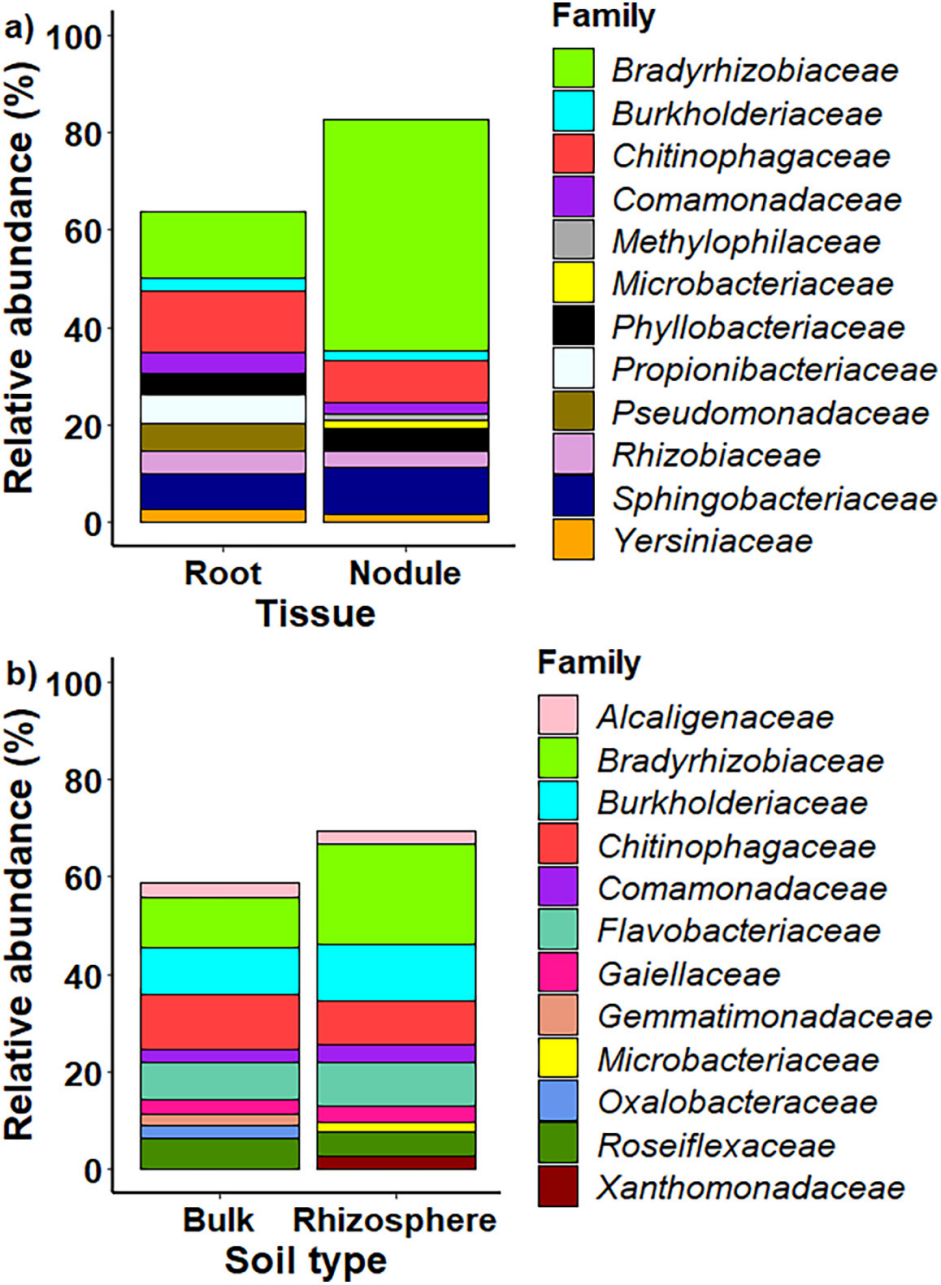
Family‐level taxonomic distribution of the endophytic bacterial communities (a) in roots and nodules and (b) soil bacterial communities in the rhizosphere and bulk soil sampled from 10 invasion sites of the legume *Lupinus polyphyllus* . Shown are the 10 most abundant families per tissue and soil type. Data are pooled across the 10 invasion sites.

Root and nodule samples collected from 10 established invasions of *L. polyphyllus* yielded a total of 13,478 bacterial ZOTUs that were assigned to 28 phyla, 66 classes, 120 orders and 310 families. The main bacterial phylum in both roots and nodules was *Proteobacteria* (58% of ZOTUs in roots and 74% in nodules), followed by *Bacteroidetes* (22% and 20%, respectively) and *Actinobacteria* (11% and 3%, respectively). In both plant tissue types, the top three bacterial families were *Bradyrhizobiaceae*, *Chitinophagaceae* and *Sphingobacteriaceae*, with *Chitinophagaceae* being the most abundant family in roots and *Bradyrhizobiaceae* being the most abundant family in nodules (Figure 1a).

**FIGURE 2:**
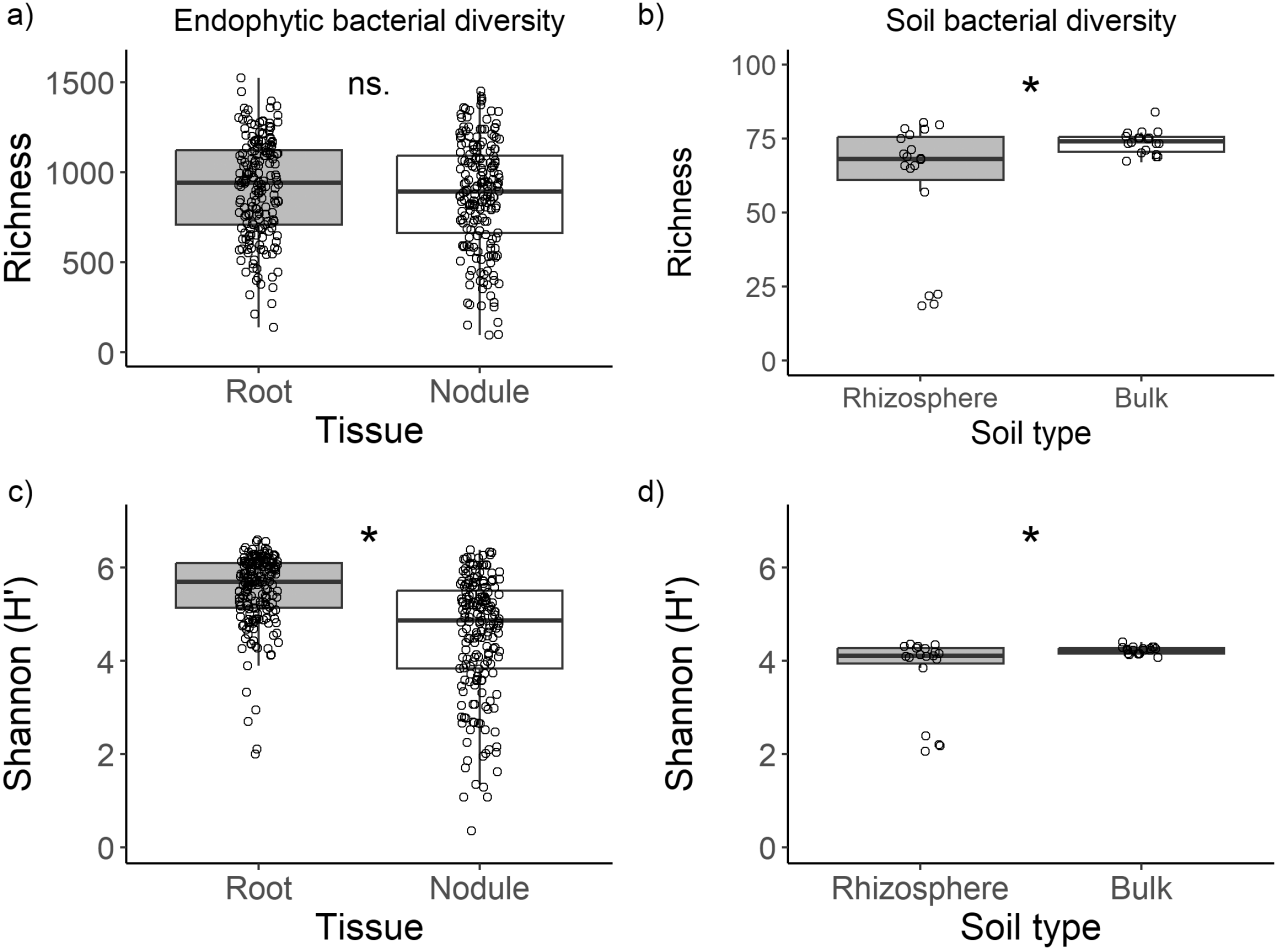
(a, c) Plant endophytic and (b, d) soil bacterial alpha diversity based on 10 invasion sites of the legume *Lupinus polyphyllus*. Shown are the medians, the lower and upper quartiles (boxes), the minimum and maximum values (whiskers) and individual raw data points. Significant differences based on LMMs are indicated with an asterisk. ns. = non‐significant.

**FIGURE 3:**
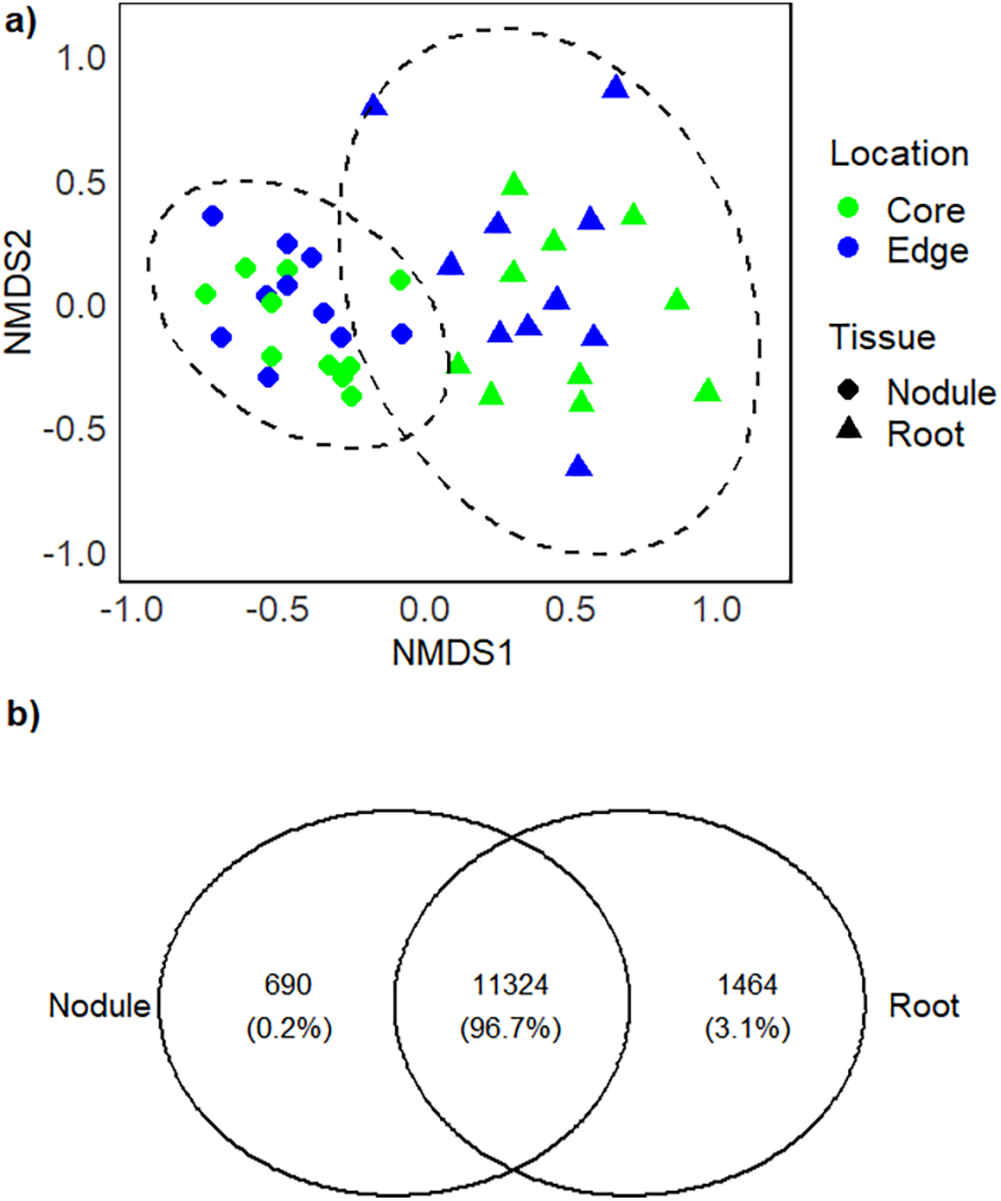
(a) Non‐metric multidimensional scaling (NMDS) ordination of the endophytic bacterial communities of roots and nodules based on 10 invasion sites of the legume *Lupinus polyphyllus*. Convex hulls are drawn around root and nodule samples. Each symbol is based on 10 plants averaged per location within each invasion site. (b) Venn diagram showing the number of unique and shared ZOTUs with their relative abundances in roots and nodules.

Measured as ZOTU richness, the alpha diversity of endophytic bacteria was similar between plant tissue types and sampling locations within invasion sites (Table 1, Figure 2a). By contrast, measures of Shannon diversity were similar between core and edge locations, but differed between tissue types (Table 1), being 24% higher in roots than in nodules (Figure 2c). Likewise, the composition of endophytic bacterial communities differed between plant tissue types but not between core and edge locations within invasion sites (Table 2, Figure 3a, Figure S2). Community composition of nodules exhibited a smaller variance than those of roots (*F*_1,38_ = 28.500, *p* < 0.001), meaning that they clustered more tightly and were thus more homogeneous (Figure 3a). The variances of endophytic bacterial communities did not differ between core and edge locations within invasions (*F*_1,38_ = 0.223, *p* = 0.639).

**TABLE 1:** Results of linear mixed models analysing alpha diversity based on endophytic and soil bacterial ZOTUs sampled in core and edge locations within 10 invasion sites of the legume *Lupinus polyphyllus* .

Data	Fixed factors	Richness		Shannon (H′)	
		*F*_df,dff_	*p*	*F*_df,dff_	*p*
Endophytic bacterial diversity	Tissue type (root, nodule)	2.107_1,367_	0.148	73.811_1,367_	**< 0.001**
	Location (core, edge)	0.447_1,368_	0.504	0.087_1,368_	0.768
	Tissue × Location	0.233_1,367_	0.629	0.048_1,367_	0.826
Soil bacterial diversity	Soil type (rhizosphere, bulk)	6.002_1,25_	0.022	5.646_1,25_	**0.025**
	Location (core, edge)	0.629_1,27_	0.435	0.831_1,27_	0.370
	Soil × Location	0.793_1,25_	0.382	0.972_1,25_	0.334

*Note:* Invasion site was used as a random factor in all models, df and ddf denote the degrees of freedom in the numerator and denominator, respectively. *p*‐values < 0.05 are in bold.

**TABLE 2:** Results of PERMANOVA analysing endophytic and soil bacterial community composition in core and edge locations within 10 invasion sites of the legume *Lupinus polyphyllus* .

Response variable	Fixed factors	df	*F*	*R* ^2^	*p*
Endophytic bacterial community	Tissue type (root, nodule)	1	11.334	0.231	**0.001**
	Location (core, edge)	1	1.257	0.026	0.190
	Tissue × Location	1	0.568	0.016	0.959
Soil bacterial community	Soil type (rhizosphere, bulk)	1	0.912	0.025	0.697
	Location (core, edge)	1	1.220	0.033	0.095
	Soil × Location	1	0.623	0.019	0.998

*Note:* Analyses are based on Bray‐Curtis dissimilarities calculated from the relative abundance of bacterial ZOTUs. *p*‐values < 0.05 are in bold.

Indicator species analysis revealed 1353 ZOTUs (10.1% of the total number) that were good indicators of roots and 1027 ZOTUs (7.6%) that were indicators of nodules. The top 10 indicator ZOTUs of roots belonged to class *Actinobacteria* and represented multiple families, while the top 10 ZOTUs of nodules belonged to the class *Alphaproteobacteria* and family *Bradyrhizobiaceae* (Table S1). Overall, 3.1% of all ZOTUs were unique to roots and 0.2% of ZOTUs were present in nodules only (Figure 3b).

### 3.2 Soil Bacterial Communities

Soil samples collected from the rhizosphere and bulk soil of the 10 established invasions yielded a total of 1141 bacterial ZOTUs that were assigned to 17 phyla, 37 classes, 69 orders and 141 families. The main bacterial phyla in both soil types were *Proteobacteria*, *Bacteroidetes* and *Actinobacteria*, with the top three families being *Bradyrhizobiaceae*, *Burkholderiaceae* and *Chitinophagaceae* (Figure 1b). The relative proportions of these top families differed between soil types, with *Bradyrhizobiaceae* being most abundant in the rhizosphere and *Chitinophagaceae* in bulk soil (Figure 1b).

Both measures of bacterial alpha diversity (ZOTU richness and Shannon diversity) differed between soil types (Table 1), with bulk soil containing 27% more bacterial ZOTUs and having 12% higher ZOTU diversity than the rhizosphere soil (Figure 2b,d). However, the rhizosphere soil samples from four invasion sites had remarkably low alpha diversity (Figure 2b,d), and when these samples were excluded from the analysis, there was no difference in ZOTU richness or Shannon diversity between soil types (*F*_1,23_ = 1.733, *p* = 0.201 and *F*_1,23_ = 1.851, *p* = 0.187, respectively). Sampling location did not explain variation in soil bacterial alpha diversity either on its own or through an interaction with soil type (Table 1). There was no difference in the composition of soil bacterial communities between rhizosphere and bulk soil or between core and edge locations within invasion sites (Table 2, Figures S2 and S3). Moreover, the variances of the soil bacterial communities were similar between the two soil types (*F*_1,36_ = 0.999, *p* = 0.324) and sampling locations (*F*_1,36_ = 0.640, *p* = 0.429).

**FIGURE 4:**
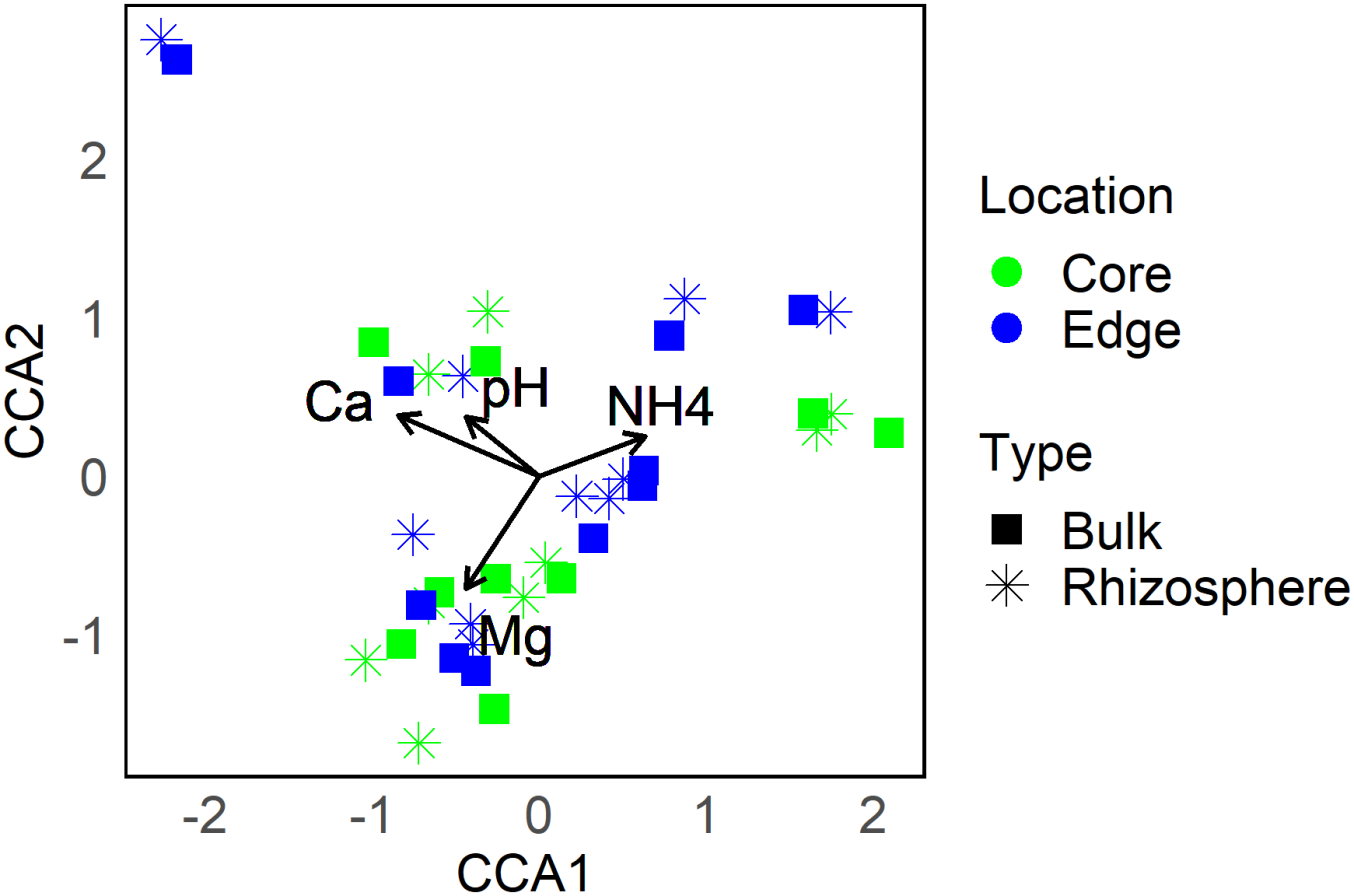
Canonical correspondence analysis (CCA) ordination of soil bacterial communities based on 10 invasion sites of the legume *Lupinus polyphyllus* . Arrows show vectors of significant soil variables (*p* < 0.05). Colours represent soil samples from core and edge locations within each invasion site. Each symbol is based on 3 pooled samples per location within each site.

Canonical correspondence analysis (CCA) revealed that the soil bacterial communities were only weakly associated with soil chemistry (*N*, NH_4_^+^, P, Ca, Mg, pH) that altogether explained 2.2% of the bacterial composition variation (*R* ^2^_Adj_ = 2.2%). Among the six soil variables considered, NH_4_^+^, Ca and pH correlated with the first CCA axis, while Mg correlated with the second CCA axis, and P and total *N* were not associated with the soil bacterial communities (Table S2, Figure 4).

### 3.3 Soil Nutrient Content and pH

Soil in the core locations within invasion sites tended to have higher total *N* content compared to invasion edges, although this difference was only marginally significant (Table 3). For the other nutrients considered (NH_4_^+^, P, Ca, Mg) and pH, there was no difference between core and edge locations within invasion sites (Table 3).

**TABLE 3:** Results of *t*‐tests comparing soil chemistry between core and edge locations within 10 invasion sites of the legume *Lupinus polyphyllus* (*n* = 20 soil samples).

Variable	Core (mean ± SD)	Edge (mean ± SD)	*t*	*p*
log(total *N*)	3.75 ± 2.31	2.55 ± 2.62	1.784	0.091
log(NH_4_^+^)	0.57 ± 0.29	0.46 ± 0.12	1.146	0.267
log(P)	9.75 ± 8.64	9.27 ± 9.467	0.425	0.676
log(Ca)	1393 ± 791	1363 ± 1198	0.364	0.720
Mg	245.30 ± 133.30	189.80 ± 135.60	0.923	0.368
pH	5.99 ± 0.41	5.84 ± 0.49	0.743	0.467

*Note:* All nutrients are reported as mg/L.

## 4 Discussion

We explored plant endophytic and soil bacterial communities in core and edge locations within 10 established invasions of the legume *L. polyphyllus* in southwestern Finland, all of which have existed for more than 10 years. We found that the Shannon diversity and composition of endophytic bacterial communities differed between roots and nodules but not between core and edge locations within invasion sites. Specifically, roots had 24% higher Shannon diversity than nodules, which were instead characterised by more‐homogenous bacterial communities dominated by rhizobial ZOTUs in family *Bradyrhizobiaceae*. In soils invaded by *L. polyphyllus* , the alpha diversity of the bacterial communities was shaped by soil type, with bulk soil having 27% higher bacterial richness and 12% higher Shannon diversity than the rhizosphere. However, this result was driven by four influential rhizosphere samples and disappeared when these samples were excluded from the analysis. The composition of soil bacterial communities did not differ between rhizosphere and bulk soil or between core and edge locations within an invasion site but was weakly associated with soil chemistry (NH_4_^+^, Ca, Mg, pH). Moreover, soil variables were similar between core and edge locations.

In these endophytic and soil bacterial communities, the lack of a difference between core and edge locations within the established invasions of *L. polyphyllus* indicates that this plant invader may not induce dramatic changes in the plant and soil microbiota over the course of a local invasion. This finding is consistent with the results of our previous study conducted at the same sites of *L. polyphyllus* , in which we observed no overall difference in the rhizosphere bacterial communities between invaded and uninvaded soils (Mousavi and Ramula 2024). Although several studies have reported changes in plant and/or soil microbiota associated with plant invasions (reviewed in Custer and van Diepen 2020; Torres et al. 2021), negligible effects similar to ours are not exceptional (Custer and van Diepen 2020). For example, for five Australian legumes, N‐fixing bacterial communities in nodules and associated soils rarely differed between invasive and non‐invasive populations (Birnbaum et al. 2016). Similarly, a study of the perennial wetland plant *Phragmites australis* in Michigan (USA) found that native and non‐native populations harboured identical root microbial communities (bacteria and fungi) (Bickford et al. 2018). It is also possible, however, that the lack of differences here in the endophytic and soil bacterial communities between core and edge locations within lupin invasions could be also explained by uncertainty related to our sampling design. We assumed that invasion edges with lower invader density represented invasion fronts, but this assumption may not hold if the invasions were declining rather than expanding. The present growth status of the studied invasions is not known, but demographic models parameterised using data from the years 2010 to 2011 predicted that most of them were either stable or increasing over time (asymptotic population growth rates, *λ*: 1.008–3.198), with only one population predicted to decline (*λ* = 0.739; Ramula 2014). Moreover, rhizosphere soil in core locations within invasion sites tended to have a slightly higher *N* content than edges–a trend that could be due to a longer history of rhizobia in the core locations, and which would support the idea that the sampled edges indeed represented invasion fronts. Finally, as we focused on the bacterial communities only, we cannot rule out the possibility that there might have been differences in the diversity or composition of fungal communities between core and edge locations within invasions. However, soil fungal communities in general tend to be less responsive to plant invasions than soil bacterial communities (reviewed in Torres et al. 2021), and this is especially likely to be true for our study species, which is well known to form mutualistic associations with symbiotic N‐fixing bacteria, while the nature of its mycorrhizal associations (if any) are still being debated (Oba, Tawaray, and Wagatsuma 2001; Shi et al. 2017).

Plant endophytic microbiomes are typically influenced by tissue type (e.g., Given et al. 2020; Trivedi et al. 2020), which often results in distinct microbial community structures in different plant organs (Trivedi et al. 2020). As we predicted, the roots and nodules of *L. polyphyllus* were inhabited by unique endophytic bacterial communities, with putative N‐fixing bacteria (phylum *Proteobacteria*, family *Bradyrhizobiaceae*) being predominant in nodules. Due to this dominance, bacterial alpha diversity (Shannon diversity) was higher in roots than in nodules, but there was no difference in overall bacterial richness (i.e., the number of ZOTUs) between roots and nodules. The nodules we examined also contained other putatively non‐N‐fixing bacteria, as has been reported previously for other legumes (De Meyer, De Beuf, and Willems 2015; Birnbaum et al. 2016), including the study species (Ramula, Mousavi, and Kalske 2023). In the present study, these other top indicator ZOTUs found in the nodules represented the families *Chromobacteriaceae*, *Brucellaceae*, and *Bartonellaceae* (Table S1). The type genera of these three families contain pathogenic bacteria. *Chromobacterium* includes opportunistic pathogenic species (Loke and Saud 2021), *Brucella* spp. are well‐known animal and human pathogens (Kämpfer, Wohlgemuth, and Scholz 2014), and *Bartonella* spp. are recognised as (mostly) animal pathogens (Baldani, Santos, and Massard 2014). Here, the roots of *L. polyphyllus* were dominated by members of the family *Chitinophagaceae* that have been reported to promote plant growth (Madhaiyan et al. 2015) as well as to protect against plant pathogens (Cárrion et al. 2019). In addition to plant tissue type, plant endophytic microbiomes are often shaped by plant genotype (e.g., Bulgarelli et al. 2013; Wagner et al. 2016). Previous studies have found that genotype has a much smaller effect on root‐associated microbes than on leaf‐associated microbes (Wagner et al. 2016; Dastogeer et al. 2020), while for the annual legume, *Medicago truncatula* , genotype modulated the root endosphere community in particular (Brown et al. 2020). As we did not consider genotype in the present field‐based study, our interpretation of the factors that structure plant microbiomes is somewhat limited.

We observed that soil bacterial alpha diversity (in terms of both ZOTU richness and Shannon diversity) was higher in bulk soil than in the rhizosphere soil associated with the plant invader when all soil samples were included, but there was no difference in the community composition of bacteria between the two soil types. This result agrees with previous findings from different study systems that have reported microbial diversity to be highest in bulk soil and to decline moving through the rhizosphere to the inside of the root (reviewed in Reinhold‐Hurek et al. 2015). Nevertheless, the difference we detected in alpha diversity between soil types disappeared when four rhizosphere samples with low diversity were excluded from the data. It should be noted, though, that our diversity measures of endophytic and soil bacteria are not directly comparable to each other due to the different laboratory protocols used. Soil samples were not adequately diluted, which resulted in much lower bacterial diversity compared to the root and nodule samples. Here, the soil bacterial community structure was weakly associated with soil chemistry (NH_4_^+^, Ca, Mg, pH), with these variables explaining only 2.2% of the variation. Both rhizosphere and bulk soils in the present study were dominated by members of the families *Bradyrhizobiaceae*, *Burkholderiaceae* and *Chitinophagaceae*, while in our previous study based on the same study sites, the top three families in the rhizosphere of *L. polyphyllus* were *Chitinophagaceae*, *Bradyrhizobiaceae* and *Gemmatimonaceae* (Mousavi and Ramula 2024). Similar to the endophytic bacterial communities, soil bacterial communities did not differ between core and edge locations within established invasions in terms of alpha diversity or composition. The same was true also for the nutrient content and pH of rhizosphere soil, which were very similar between core and edge locations. The only exception was total *N* content, which tended to be slightly higher in core locations compared to edges. This result makes sense given the N‐fixing ability of *L. polyphyllus* , which would be predicted to increase soil N content (Hiltbrunner et al. 2014). Indeed, a previous study revealed an increase in soil nitrate, ammonium and phosphate contents in more established (≥ 15 years) invasions of the species compared to younger invasions (≤ 10 years) in Finland (Prass et al. 2022).

## 5 Conclusions

Our results reveal that the root, nodule and soil bacterial communities associated with *L. polyphyllus* in southwestern Finland are dominated by a few bacterial phyla that are also common for other (non‐invasive) plant species. Moreover, the endophytic bacterial communities of roots and nodules are distinct from each other in terms of both alpha (Shannon) diversity and composition. Nodules contain predominantly N‐fixing bacteria (47.7%) as well as other putatively non‐N‐fixing bacterial families. Overall, the root, nodule and soil bacterial communities are homogeneous within established invasions of *L. polyphyllus* (> 10 years old), indicating that this plant invader may not induce dramatic changes in the bacterial communities with which it associates over the course of the local invasion process. Moreover, these bacterial communities may not be critical to the local invasion success of the species.

## Author Contributions

**Satu Ramula:** conceptualization (lead), data curation (equal), formal analysis (lead), funding acquisition (lead), investigation (lead), methodology (lead), project administration (lead), visualization (lead), writing – original draft (lead). **Seyed Abdollah Mousavi:** data curation (equal), formal analysis (supporting), methodology (supporting), visualization (supporting), writing – review and editing (supporting). **Eero J. Vesterinen:** conceptualization (supporting), data curation (equal), writing – review and editing (supporting).

## Conflicts of Interest

EJV owns the company Bioname.

## Supporting information


Data S1.

## Data Availability

The raw data of root and nodule samples were deposited in the NCBI Sequence Read Archive (SRA) database under BioProject: PRJNA1121206 (accession no. SRR29346803‐SRR29347200), and the raw data of soil samples were deposited in the NCBI SRA under BioProject: PRJNA1121614 (accession no. SRR29330554‐SRR29330593). The data used for the statistical analyses were deposited in the Dryad repository https://doi.org/10.5061/dryad.qz612jmrc.

## References

[ece370669-bib-0001] Baldani, C. D. , H. A. Santos , and C. L. Massard . 2014. “The Family Bartonellaceae.” In The Prokaryotes, edited by E. Rosenberg , E. F. De Long , S. Lory , E. Stackebrandt , and F. Thompson , 81–114. Berlin Heidelberg: Springer‐Verlag.

[ece370669-bib-0002] Batten, K. M. , K. M. Scow , K. F. Davies , and S. P. Harrison . 2006. “Two Invasive Plants Alter Soil Microbial Community Composition in Serpentine Grasslands.” Biological Invasions 8: 217–230.

[ece370669-bib-0003] Begum, N. , C. Qin , M. A. Ahanger , et al. 2019. “Role of Arbuscular Mycorrhizal Fungi in Plant Growth Regulation: Implications in Abiotic Stress Tolerance.” Frontiers in Plant Science 10: 1068. 31608075 10.3389/fpls.2019.01068 PMC6761482

[ece370669-bib-0004] Bickford, W. A. , D. E. Goldberg , K. P. Kowalski , and D. R. Zak . 2018. “Root Endophytes and Invasiveness: No Difference Between Native and Non‐Native *Phragmites* in the Great Lakes Region.” Ecosphere 9: e02526.

[ece370669-bib-0005] Birnbaum, C. , A. Bissett , P. H. Thrall , and M. R. Leishman . 2016. “Nitrogen‐Fixing Bacterial Communities in Invasive Legume Nodules and Associated Soils Are Similar Across Introduced and Native Range Populations in Australia.” Journal of Biogeography 43: 1631–1644.

[ece370669-bib-0006] Brown, S. P. , M. A. Grillo , J. C. Podowski , and K. D. Heath . 2020. “Soil Origin and Plant Genotype Structure Distinct Microbiome Compartments in the Model Legume *Medicago truncatula* .” Microbiome 8: 139. 32988416 10.1186/s40168-020-00915-9 PMC7523075

[ece370669-bib-0007] Bulgarelli, D. , K. Schlaeppi , S. Spaepen , V. Loren , E. V. L. van Themaat , and P. Schulze‐Lefert . 2013. “Structure and Functions of the Bacterial Microbiota of Plants.” Annual Review of Plant Biology 64: 807–838. 10.1146/annurev-arplant-050312-120106 23373698

[ece370669-bib-0008] Buswell, J. M. , A. T. Moles , and S. Hartley . 2011. “Is Rapid Evolution Common in Introduced Plant Species?” Journal of Ecology 99: 214–224.

[ece370669-bib-0009] Cárrion, V. J. , J. Perez‐Jaramillo , V. Cordovez , et al. 2019. “Pathogen‐Induced Activation of Disease‐Suppressive Functions in the Endophytic Root Microbiome.” Science 366: 606–612. 31672892 10.1126/science.aaw9285

[ece370669-bib-0010] Chelius, M. K. , and E. W. Triplett . 2001. “The Diversity of Archaea and Bacteria in Association With the Roots of *Zea mays* L.” Microbial Ecology 41: 252–263. 11391463 10.1007/s002480000087

[ece370669-bib-0011] Clements, D. R. , and V. L. Jones . 2021. “Rapid Evolution of Invasive Weeds Under Climate Change: Present Evidence and Future Research Needs.” Frontiers in Agronomy 3: 664034.

[ece370669-bib-0012] Colautti, R. I. , and S. C. H. Barrett . 2013. “Rapid Adaptation to Climate Facilitates Range Expansion of an Invasive Plant.” Science 342: 364–366. 24136968 10.1126/science.1242121

[ece370669-bib-0013] Compant, S. , C. Clément , and A. Sessitsch . 2010. “Plant Growth‐Promoting Bacteria in the Rhizo‐ and Endosphere of Plants: Their Role, Colonization, Mechanisms Involved and Prospects for Utilization.” Soil Biology and Biochemistry 42: 669–678.

[ece370669-bib-0014] Custer, G. F. , and L. T. van Diepen . 2020. “Plant Invasion Has Limited Impact on Soil Microbial *α*‐Diversity: A Meta‐Analysis.” Diversity 12: 112.

[ece370669-bib-0015] Dastogeer, K. M. G. , F. H. Tumpa , A. Sultana , M. A. Akter , and A. Chakraborty . 2020. “Plant Microbiome–An Account of the Factors That Shape Community Composition and Diversity.” Current Plant Biology 23: 100161.

[ece370669-bib-0016] Dawson, W. , and M. Schrama . 2016. “Identifying the Role of Soil Microbes in Plant Invasions.” Journal of Ecology 104: 1211–2118.

[ece370669-bib-0017] De Cáceres, M. , and P. Legendre . 2009. “Associations Between Species and Groups of Sites: Indices and Statistical Inference.” Ecology 90: 3566–3574. 20120823 10.1890/08-1823.1

[ece370669-bib-0018] De Meyer, S. E. , K. De Beuf , and A. Willems . 2015. “A Large Diversity of Non‐rhizobial Endophytes Found in Legume Root Nodules in Flanders (Belgium).” Soil Biology and Biochemistry 83: 1–11.

[ece370669-bib-0019] Eckstein, R. L. , E. Welk , Y. P. Klinger , et al. 2023. “Biological Flora of Central Europe—*Lupinus polyphyllus* Lindley.” Perspectives in Plant Ecology, Evolution and Systematics 58: 125715.

[ece370669-bib-0020] Edgar, R. C. 2010. “Search and Clustering Orders of Magnitude Faster Than BLAST.” Bioinformatics 26: 2460–2461. 20709691 10.1093/bioinformatics/btq461

[ece370669-bib-0021] Ghyselinck, J. , S. Pfeiffer , K. Heylen , A. Sessitch , and P. De Vos . 2013. “The Effect of Primer Choice and Short Read Sequences on the Outcome of 16S rRNA Gene Based Diversity Studies.” PLoS One 8: e71360. 23977026 10.1371/journal.pone.0071360 PMC3747265

[ece370669-bib-0022] Gibbons, S. M. , Y. Lekberg , D. L. Mummey , N. Sangwan , P. W. Ramsey , and J. A. Gilbert . 2017. “Invasive Plants Rapidly Reshape Soil Properties in a Grassland Ecosystem.” mSystems 2: e00178‐16. 28289729 10.1128/mSystems.00178-16 PMC5340861

[ece370669-bib-0023] Given, C. , E. Häikiö , M. Kumar , and R. Nissinen . 2020. “Tissue‐Specific Dynamics in the Endophytic Bacterial Communities in Arctic Pioneer Plant *Oxyria digyna* .” Frontiers in Plant Science 11: 561. 32528486 10.3389/fpls.2020.00561 PMC7247849

[ece370669-bib-0024] Hiltbrunner, E. , R. Aerts , T. Buhlmann , et al. 2014. “Ecological Consequences of the Expansion of N_2_‐Fixing Plants in Cold Biomes.” Oecologia 176: 11–24. 24938834 10.1007/s00442-014-2991-x

[ece370669-bib-0025] Jara‐Servin, A. , A. Silva , H. Barajas , R. Cruz‐Ortega , C. Tinoco‐Ojanguren , and L. D. Alcaraz . 2023. “Root Microbiome Diversity and Structure of the Sonoran Desert Buffelgrass ( *Pennisetum ciliare* L.).” PLoS One 18: e0285978. 37205698 10.1371/journal.pone.0285978 PMC10198571

[ece370669-bib-0026] Kämpfer, P. , S. Wohlgemuth , and H. Scholz . 2014. “The Family Brucellaceae.” In The Prokaryotes, edited by E. Rosenberg , E. F. De Long , S. Lory , E. Stackebrandt , and F. Thompson , 155–178. Berlin Heidelberg: Springer‐Verlag.

[ece370669-bib-0027] Kankaanpää, T. , E. Vesterinen , B. Hardwick , et al. 2020. “Parasitoids Indicate Major Climate‐Induced Shifts in Arctic Communities.” Global Change Biology 26: 6276–6295. 32914511 10.1111/gcb.15297 PMC7692897

[ece370669-bib-0028] Kaunisto, K. M. , T. Roslin , M. R. Forbes , et al. 2020. “Threats From the Air: Damselfly Predation on Diverse Prey Taxa.” Journal of Animal Ecology 89: 1365–1374. 32124439 10.1111/1365-2656.13184

[ece370669-bib-0029] Kuznetsova, A. , P. B. Brockhoff , and R. H. Christensen . 2017. “lmerTest Package: Tests in Linear Mixed Effects Models.” Journal of Statistical Software 82: 1–26.

[ece370669-bib-0030] Lane, D. J. 1991. “16S/23S rRNA sequencing.” In Nucleic Acid Techniques in Bacterial Systematics, edited by E. Stackebrandt and M. Goodfellow , 115–175. NewYork: Wiley.

[ece370669-bib-0031] Liu, C. , Y. Cui , X. Li , and M. Yao . 2021. “Microeco: An R Package for Data Mining in Microbial Community Ecology.” FEMS Microbiology Ecology 97: fiaa255. 33332530 10.1093/femsec/fiaa255

[ece370669-bib-0032] Loke, W. K. , and H. M. Saud . 2021. “Effect of *Chromobacterium violaceum* on Plant Growth‐Promoting Rhizobacteria (PGPR) Under *In‐Vitro* Conditions.” Malaysian Journal of Soil Science 25: 59–65.

[ece370669-bib-0033] Lu‐Irving, P. , J. G. Harencar , H. Sounart , et al. 2019. “Native and Invading Yellow Starthistle ( *Centaurea solstitialis* ) Microbiomes Differ in Composition and Diversity of Bacteria.” mSphere 4: e00088‐19. 30842267 10.1128/mSphere.00088-19 PMC6403453

[ece370669-bib-0034] Madhaiyan, M. , S. Poonguzhali , M. Senthilkumar , D. Pragatheswari , J.‐S. Lee , and K.‐C. Lee . 2015. “ *Arachidicoccus rhizosphaerae* Gen. Nov., Sp. Nov., a Plant‐Growth‐Promoting Bacterium in the Family *Chitinophagaceae* Isolated From Rhizosphere Soil.” International Journal of Systematic and Evolutionary Microbiology 65: 578–586. 25404481 10.1099/ijs.0.069377-0

[ece370669-bib-0035] Martin, M. 2011. “Cutadapt Removes Adapter Sequences From High‐Throughput Sequencing Reads.” EMBnet Journal 17: 10–12.

[ece370669-bib-0036] McMurdie, P. J. , and S. Holmes . 2013. “Phyloseq: An R Package for Reproducible Interactive Analysis and Graphics of Microbiome Census Data.” PLoS One 8: e61217. 23630581 10.1371/journal.pone.0061217 PMC3632530

[ece370669-bib-0037] Mousavi, S. A. , and S. Ramula . 2024. “The Invasive Legume *Lupinus polyphyllus* Has Minor Site‐Specific Impacts on the Composition of Soil Bacterial Communities.” Ecology and Evolution 14: e11030. 38357596 10.1002/ece3.11030 PMC10864723

[ece370669-bib-0038] Nentwig, W. , S. Bacher , S. Kumschick , P. Pyšek , and M. Vilá . 2018. “More Than “100 Worst” Alien Species in Europe.” Biological Invasions 20: 1611–1621.

[ece370669-bib-0039] Oba, H. , K. Tawaray , and T. Wagatsuma . 2001. “Arbuscular Mycorrhizal Colonization in *Lupinus* and Related Genera.” Soil Science and Plant Nutrition 47, no. 4: 685–694.

[ece370669-bib-0040] Oksanen, J. , G. L. Simpson , F. G. Blanchet , et al. 2022. “Vegan: Community Ecology Package.” https://CRAN.R‐project.org/package=vegan.

[ece370669-bib-0041] Parker, M. A. 2001. “Mutualism as a Constraint on Invasion Success for Legumes and Rhizobia.” Diversity and Distributions 7: 125–136.

[ece370669-bib-0042] Prass, M. , S. Ramula , M. Jauni , H. Setälä , and J. Kotze . 2022. “The Invasive Herb *Lupinus polyphyllus* Can Reduce Plant Species Richness Independently of Local Invasion Age.” Biological Invasions 24: 425–436.

[ece370669-bib-0043] R Core Team . 2023. R: A Language and Environment for Statistical Computing. Vienna, Austria: R Foundation for Statistical Computing. https://www.R‐project.org/.

[ece370669-bib-0044] Ramula, S. 2014. “Linking Vital Rates to Invasiveness of a Perennial Herb.” Oecologia 174: 1255–1264. 24390414 10.1007/s00442-013-2869-3

[ece370669-bib-0045] Ramula, S. , S. A. Mousavi , and A. Kalske . 2023. “Rhizobial Benefits to an Herbaceous Invader Depend on Context and Symbiotic Strain.” Plant and Soil 490: 603–616.

[ece370669-bib-0046] Reinhold‐Hurek, B. , W. Bunger , C. S. Burbano , R. Sabale , and T. Hurek . 2015. “Roots Shaping Their Microbiome: Global Hotspots for Microbial Activity.” Annual Review of Phytopathology 53: 403–424. 10.1146/annurev-phyto-082712-102342 26243728

[ece370669-bib-0047] Rognes, T. , T. Flouri , B. Nichols , C. Quince , and F. Mahé . 2016. “VSEARCH: A Versatile Open Source Tool for Metagenomics.” PeerJ 18: e2584. 10.7717/peerj.2584 PMC5075697 27781170

[ece370669-bib-0048] Rout, M. E. , T. H. Chrzanowski , T. K. Westlie , T. H. DeLuca , R. M. Callaway , and W. E. Holben . 2013. “Bacterial Endophytes Enhance Competition by Invasive Plants.” American Journal of Botany 100: 1726–1737. 23935109 10.3732/ajb.1200577

[ece370669-bib-0049] Shi, Z. Y. , X. L. Zhang , S. X. Xu , et al. 2017. “Mycorrhizal Relationship in Lupines: A Review.” Legume Research 40: 965–973.

[ece370669-bib-0050] Smith, S. E. , and F. A. Smith . 2011. “Roles of Arbuscular Mycorrhizas in Plant Nutrition and Growth: New Paradigms From Cellular to Ecosystem Scales.” Annual Review of Plant Biology 62: 227–250. 10.1146/annurev-arplant-042110-103846 21391813

[ece370669-bib-0051] Stępkowski, T. , J. Banasiewicz , C. E. Granada , M. Andrews , and L. M. Passaglia . 2018. “Phylogeny and Phylogeography of Rhizobial Symbionts Nodulating Legumes of the Tribe Genisteae.” Genes 9: 163. 29538303 10.3390/genes9030163 PMC5867884

[ece370669-bib-0052] Stepkowski, T. , C. E. Hughes , I. J. Law , et al. 2007. “Diversification of Lupine *Bradyrhizobium* Strains: Evidence From Nodulation Gene Trees.” Applied and Environmental Microbiology 73: 3254–3264. 17400786 10.1128/AEM.02125-06 PMC1907101

[ece370669-bib-0053] Torres, N. , I. Herrera , L. Fajardo , and R. O. Bustamante . 2021. “Meta‐Analysis of the Impact of Plant Invasions on Soil Microbial Communities.” BMC Ecology and Evolution 21: 1–8. 34496752 10.1186/s12862-021-01899-2 PMC8425116

[ece370669-bib-0054] Trivedi, P. , B. D. Batista , K. E. Bazany , and B. K. Singh . 2022. “Plant–Microbiome Interactions Under a Changing World: Responses, Consequences and Perspectives.” New Phytologist 234: 1951–1959. 35118660 10.1111/nph.18016

[ece370669-bib-0055] Trivedi, P. , J. E. Leach , S. G. Tringe , T. Sa , and B. K. Singh . 2020. “Plant–Microbiome Interactions: From Community Assembly to Plant Health.” Nature Reviews. Microbiology 18: 607–621. 32788714 10.1038/s41579-020-0412-1

[ece370669-bib-0056] Vesterinen, E. J. , A. I. E. Puisto , A. S. Blomberg , and T. M. Lilley . 2018. “Table for Five, Please: Dietary Partitioning in Boreal Bats.” Ecology and Evolution 8: 10914–10937. 30519417 10.1002/ece3.4559 PMC6262732

[ece370669-bib-0057] Vesterinen, E. J. , L. Ruokolainen , N. Wahlberg , et al. 2016. “What You Need Is What You Eat? Prey Selection by the Bat *Myotis daubentoniid* .” Molecular Ecology 25, no. 7: 1581–1594. 26841188 10.1111/mec.13564

[ece370669-bib-0058] Wagner, M. R. , D. S. Lundberg , T. G. Del Rio , S. G. Tringe , J. L. Dangl , and T. Mitchell‐Olds . 2016. “Host Genotype and Age Shape the Leaf and Root Microbiomes of a Wild Perennial Plant.” Nature Communications 7: 12151. 10.1038/ncomms12151 PMC4945892 27402057

[ece370669-bib-0059] Wang, X. , M. Wang , X. Xie , et al. 2020. “An Amplification‐Selection Model for Quantified Rhizosphere Microbiota Assembly.” Science Bulletin 65: 983–986. 36659026 10.1016/j.scib.2020.03.005

[ece370669-bib-0060] Zheng, D. , E. W. Alm , D. A. Stahl , and L. Raskin . 1996. “Characterization of Universal Small‐Subunit rRNA Hybridization Probes for Quantitative Molecular Microbial Ecology Studies.” Applied and Environmental Microbiology 62: 4504–4513. 8953722 10.1128/aem.62.12.4504-4513.1996 PMC168277

