# Supplementary material for: Root, Nodule and Soil Bacterial Communities Associated With the Invasive Nitrogen‐Fixing Lupinus polyphyllus
Source: Ecol Evol. 2024 Dec 5;14(12):e70669. doi: 10.1002/ece3.70669 (PMC11620983; doi:10.1002/ece3.70669)
Supplement: Supplementary file 1 — Data S1. [file ECE3-14-e70669-s001.docx]

**SUPPLEMENTARY INFORMATION**

**Root, nodule, and soil bacterial communities associated with the invasive nitrogen-fixing *Lupinus polyphyllus***

Satu Ramula^1*^, Seyed Abdollah Mousavi^1^, Eero J. Vesterinen^1^

^1^Department of Biology, University of Turku, 20014 Turku, Finland

**Correspondence:** S. Ramula, Department of Biology, University of Turku, Finland

^*^E-mail: satu.ramula@utu.fi

TABLE S1 Top ten indicator bacterial ZOTUs in the roots and nodules of the invasive legume *Lupinus polyphyllus*. The indicator value denotes the strength of the association between a ZOTU and tissue type.

| **ZOTU** | **Phylum** | **Class** | **Family** | **Indicator value** |
| --- | --- | --- | --- | --- |
| **Roots** |  |  |  |  |
| 1616 | Actinobacteria | Actinobacteria | Microbacteriaceae | 1.000 |
| 7856 | Actinobacteria | Actinobacteria | Propionibacteriaceae | 1.000 |
| 8431 | Proteobacteria | Betaproteobacteria | Burkholderiaceae | 1.000 |
| 10903 | Actinobacteria | Actinobacteria | Propionibacteriaceae | 1.000 |
| 14005 | Actinobacteria | Actinobacteria | Propionibacteriaceae | 1.000 |
| 9946 | Proteobacteria | Alphaproteobacteria | Sphingomonadaceae | 1.000 |
| 1502 | Actinobacteria | Actinobacteria | Nocardioidaceae | 1.000 |
| 4643 | Actinobacteria | Actinobacteria | Propionibacteriaceae | 1.000 |
| 7871 | Actinobacteria | Actinobacteria | Propionibacteriaceae | 1.000 |
| 6050 | Actinobacteria | Actinobacteria | Propionibacteriaceae | 0.999 |
| **Nodules** |  |  |  |  |
| 3687 | Proteobacteria | Betaproteobacteria | Chromobacteriaceae | 0.979 |
| 1126 | Proteobacteria | Alphaproteobacteria | Brucellaceae | 0.965 |
| 185 | Proteobacteria | Alphaproteobacteria | Bartonellaceae | 0.960 |
| 13485 | Proteobacteria | Alphaproteobacteria | Bradyrhizobiaceae | 0.956 |
| 13677 | Proteobacteria | Alphaproteobacteria | Bradyrhizobiaceae | 0.955 |
| 1178 | Proteobacteria | Alphaproteobacteria | Bradyrhizobiaceae | 0.951 |
| 12560 | Proteobacteria | Alphaproteobacteria | Bradyrhizobiaceae | 0.948 |
| 11823 | Proteobacteria | Alphaproteobacteria | Bradyrhizobiaceae | 0.948 |
| 1393 | Proteobacteria | Alphaproteobacteria | Bradyrhizobiaceae | 0.945 |
| 643 | Proteobacteria | Alphaproteobacteria | Bradyrhizobiaceae | 0.945 |

TABLE S2 Results of a canonical correspondence analysis (CCA) on relationships between soil chemistry and soil bacterial communities within 10 invasion sites of the legume *Lupinus polyphyllus* (n=38 bacterial samples consisting of bulk and rhizosphere soil for core and edge locations). All nutrients are reported as mg/L. *P*-values < 0.05 are in bold.

| Variable | CCA axis 1 | CCA axis 2 | *F* | *P* |
| --- | --- | --- | --- | --- |
| total N | -0.626 | -0.218 | 1.028 | 0.308 |
| NH_4_^+^ | 0.672 | 0.167 | 1.192 | **0.009** |
| P | -0.400 | 0.229 | 1.102 | 0.095 |
| Ca | -0.775 | 0.378 | 1.266 | **0.001** |
| Mg | -0.501 | -0.659 | 1.240 | **0.001** |
| pH | -0.372 | 0.454 | 1.196 | **0.004** |


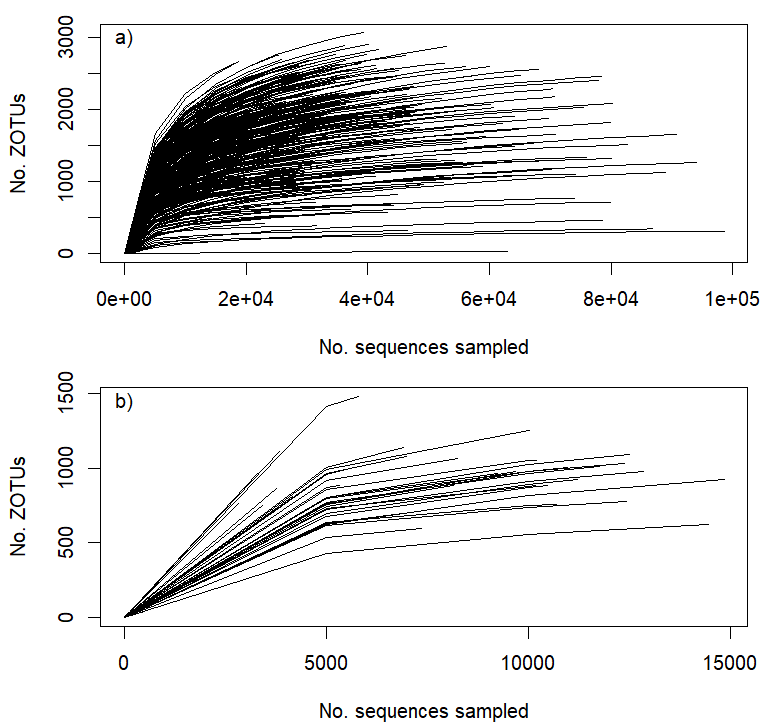


FIGURE S1 Rarefaction curves of ZOTU numbers in relation to the number of sequences sampled for root and nodule data (a) and soil data (b) of the legume *Lupinus polyphyllus.* Lines represent individual samples.

**
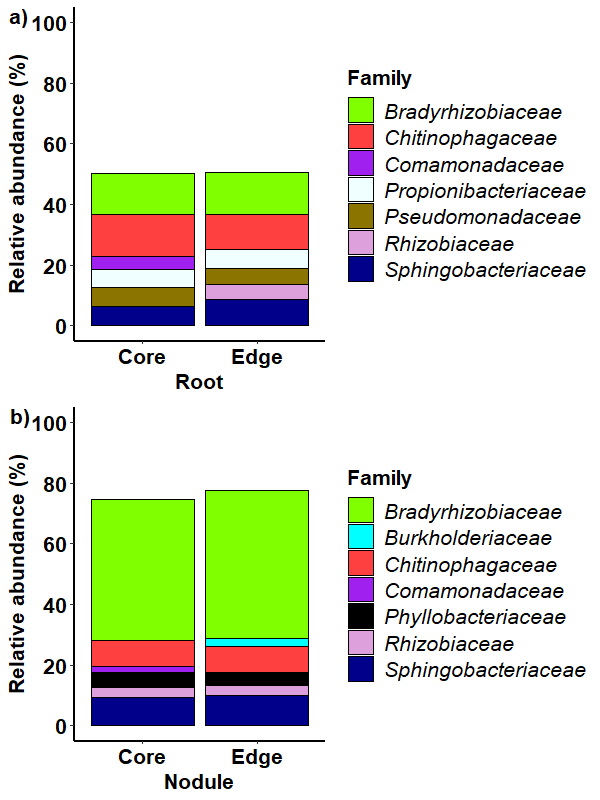
**

**
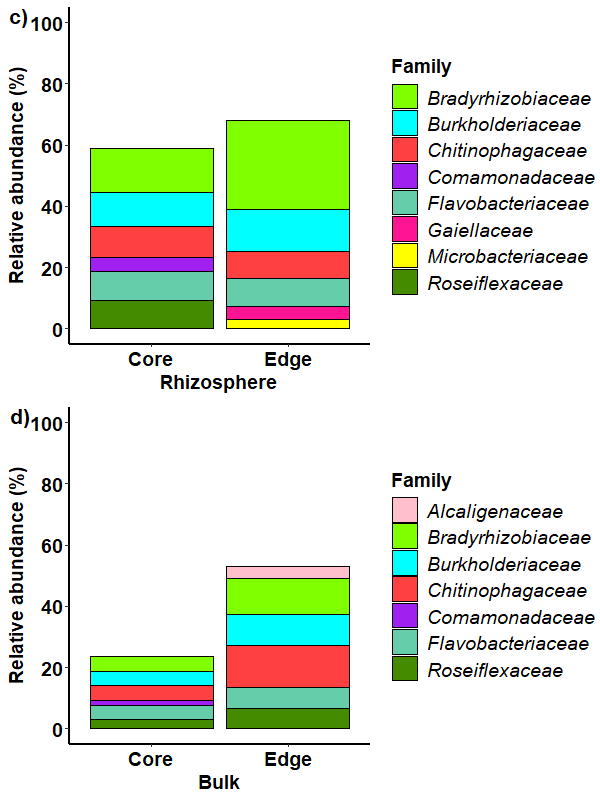
**

FIGURE S2 Family-level taxonomic distribution of the endophytic (a-b) and (c-d) soil bacterial communities in relation to core and edge locations within 10 invasion sites of the legume *Lupinus polyphyllus*. Shown are the six most abundant families, and data are pooled between plant tissue types (roots, nodule) and soil types (rhizosphere, bulk).


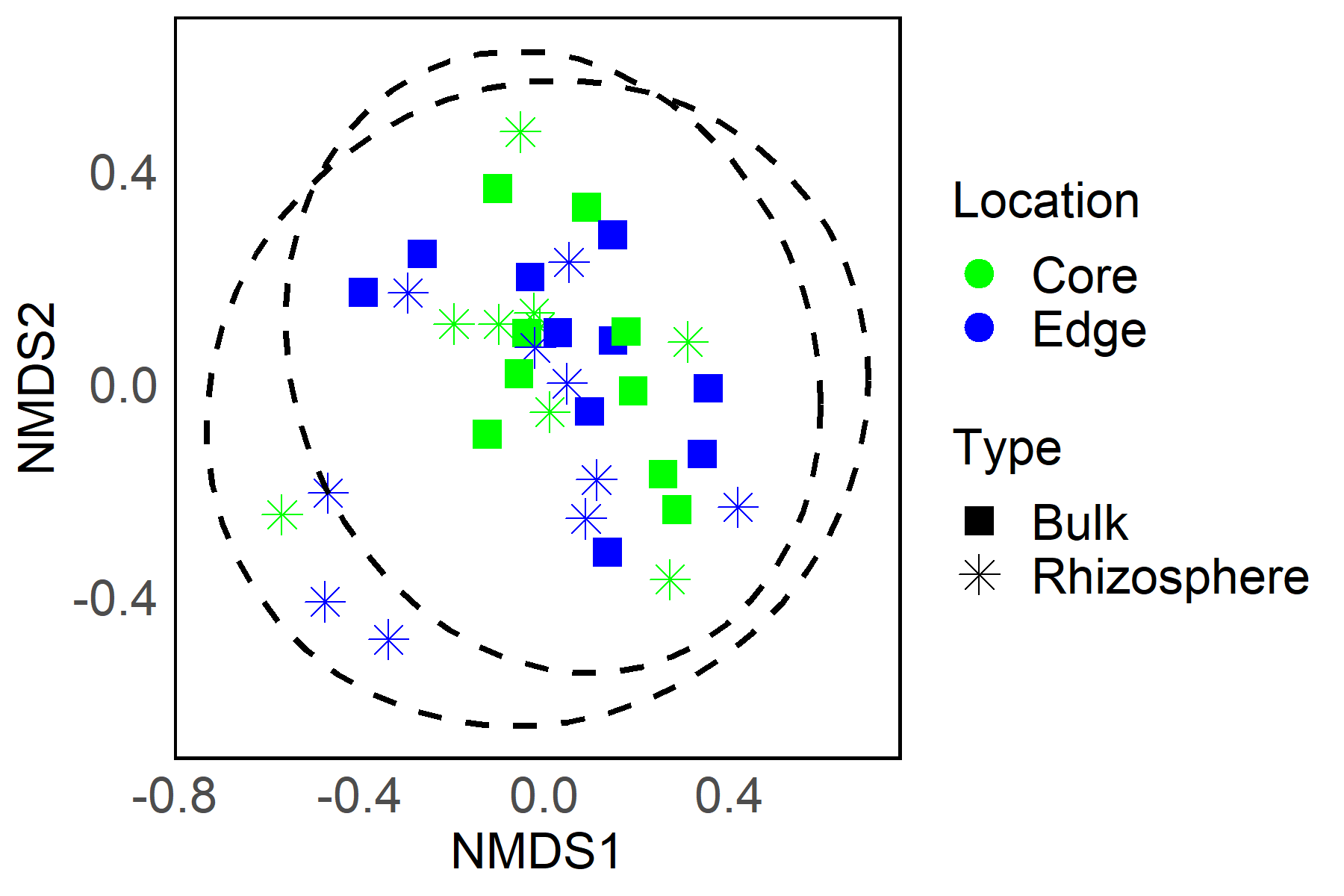


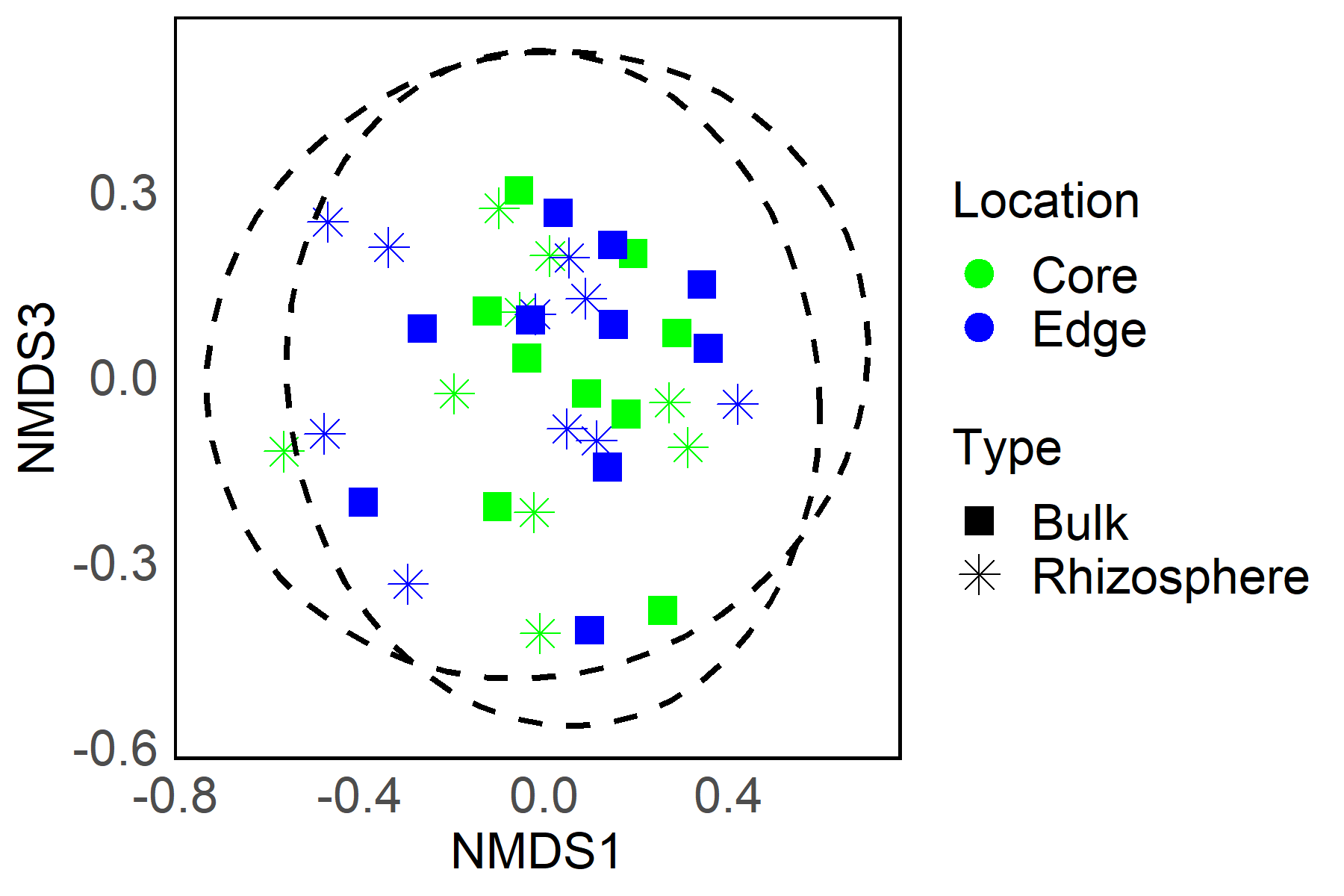


FIGURE S3 Non-metric multidimensional scaling (NMDS) ordination of soil bacterial communities based on 10 invasion sites of the legume *Lupinus polyphyllus.* Shown are three ordination axes and convex hulls are drawn around samples from core and edge locations. Each symbol is based on 3 pooled samples per location within each site.
